# Analysis of the gut microbiome and plasma short-chain fatty acid profiles in a spontaneous mouse model of metabolic syndrome

**DOI:** 10.1038/s41598-017-16189-5

**Published:** 2017-11-20

**Authors:** Kazuchika Nishitsuji, Jinzhong Xiao, Ryosuke Nagatomo, Hitomi Umemoto, Yuki Morimoto, Hiroyasu Akatsu, Koichi Inoue, Koichi Tsuneyama

**Affiliations:** 1 Department of Molecular Pathology, Graduate School of Biomedical Sciences, Tokushima University, 3-18-15 Kuramoto-cho, Tokushima, 770-8503 Japan; 2 Next Generation Science Institute, Morinaga Milk Industry Co., Ltd., 5-1-83 Higashihara, Zama, Kanagawa 252-8583 Japan; 3 Laboratory of Clinical and Analytical Chemistry, College of Pharmaceutical Sciences, Ritsumeikan University, 1-1-1 Nojihigashi, Kusatsu, Shiga 525-8577 Japan; 4 Education Support Room for Anatomy, Tokushima University, 3-18-15 Kuramoto-cho, Tokushima, 770-8503 Japan; 5 Department of Pathology and Laboratory Medicine, Graduate School of Biomedical Sciences, Tokushima University, 3-18-15 Kuramoto-cho, Tokushima, 770-8503 Japan; 6 Department of Medicine for Aging in Place and Community-Based Medical Education, Nagoya City University, Graduate School of Medical Sciences, 1 Kawasumi, Mizuho-cho, Mizuho-ku, Nagoya, Aichi 467-8601 Japan

## Abstract

Male Tsumura Suzuki obese diabetes (TSOD) mice spontaneously develop obesity and obesity-related metabolic syndrome. Gut dysbiosis, an imbalance of gut microbiota, has been implicated in the pathogenesis of metabolic syndrome, but its mechanisms are unknown. Short-chain fatty acids (SCFAs) are the main fermentation products of gut microbiota and a link between the gut microbiota and the host’s physiology. Here, we investigated a correlation among gut dysbiosis, SCFAs, and metabolic syndrome in TSOD mice. We detected enriched levels of Gram-positive bacteria and corresponding decreases in Gram-negative bacteria in 24-wk-old metabolic syndrome-affected TSOD mice compared with age-matched controls. The abundance of Bacteroidetes species decreased, the abundance of Firmicutes species increased, and nine genera of bacteria were altered in 24-wk-old TSOD mice. The total plasma SCFA level was significantly lower in the TSOD mice than in controls. The major plasma SCFA—acetate—decreased in TSOD mice, whereas propionate and butyrate increased. TSOD mice had no minor SCFAs (valerate and hexanoate) but normal mice did. We thus concluded that gut dysbiosis and consequent disruptions in plasma SCFA profiles occurred in metabolic syndrome-affected TSOD mice. We also propose that the TSOD mouse is a useful model to study gut dysbiosis, SCFAs, and metabolic syndrome.

## Introduction

Metabolic syndrome comprises a combination of obesity-related metabolic alterations that increases the risk of type 2 diabetes mellitus and cardiovascular disease^1,2^. The clinical hallmarks of this syndrome include insulin resistance, hyperglycemia, hyperlipidemia, and nonalcoholic fatty liver disease (NAFLD)^3,4^. The essential feature of metabolic syndrome is a state of low-grade inflammation^5^. Gut microbiota has been implicated as a pathogenic factor that affects a host’s metabolism^6^. Mammals harbor diverse and immensely active gut microbiota that consists of more than 10 trillion microbial cells and more than 1000 microbial strains^7^. Via dynamic crosstalk with a host, this commensal microbiota can have a number of functions that affect the host’s physiology, from immune responses to energy metabolism^8,9^. Growing evidence supports the belief that gut microbiota is closely involved in the development of various diseases, including chronic gastrointestinal diseases^10^, neurological diseases^11,12^, and systemic diseases^13,14^. Alterations in gut microbiota composition may play a critical role in the development of metabolic syndrome, which is especially relevant to obesity-associated inflammation (i.e., diabetes mellitus, NAFLD, and nonalcoholic steatohepatitis [NASH])^15–27^. The mechanism by which gut microbiota affects a host’s physiology may be at least partly mediated by short-chain fatty acids (SCFAs), which contain 1–6 carbons and are the most abundant product of bacterial fermentation of undigested dietary fibers^8,28–30^. SCFAs can activate G-coupled receptors, inhibit histone deacetylase (HDAC), and be used as an energy substrate, thereby affecting the host’s physiological processes^30^.

Tsumura Suzuki obese diabetes (TSOD) mice were originally established as a spontaneous model of type 2 diabetes mellitus^31,32^ and were also shown to spontaneously develop NASH^33^, a progressive phenotype of NAFLD^34^. Given that these pathological manifestations in TSOD mice are closely related to low-grade inflammation, we hypothesized that alterations in gut microbiota and plasma SCFA profiles in TSOD mice may affect a host’s immune system and induce inflammation, which would underlie the development of metabolic syndrome in TSOD mice. In this study, we analyzed the gut microbiome and plasma SCFA profiles in 24-wk-old TSOD mice that had already developed insulin resistance and NASH^33^.

## Results

### Analysis of the gut microbiome in TSOD mice

**Figure 1:**
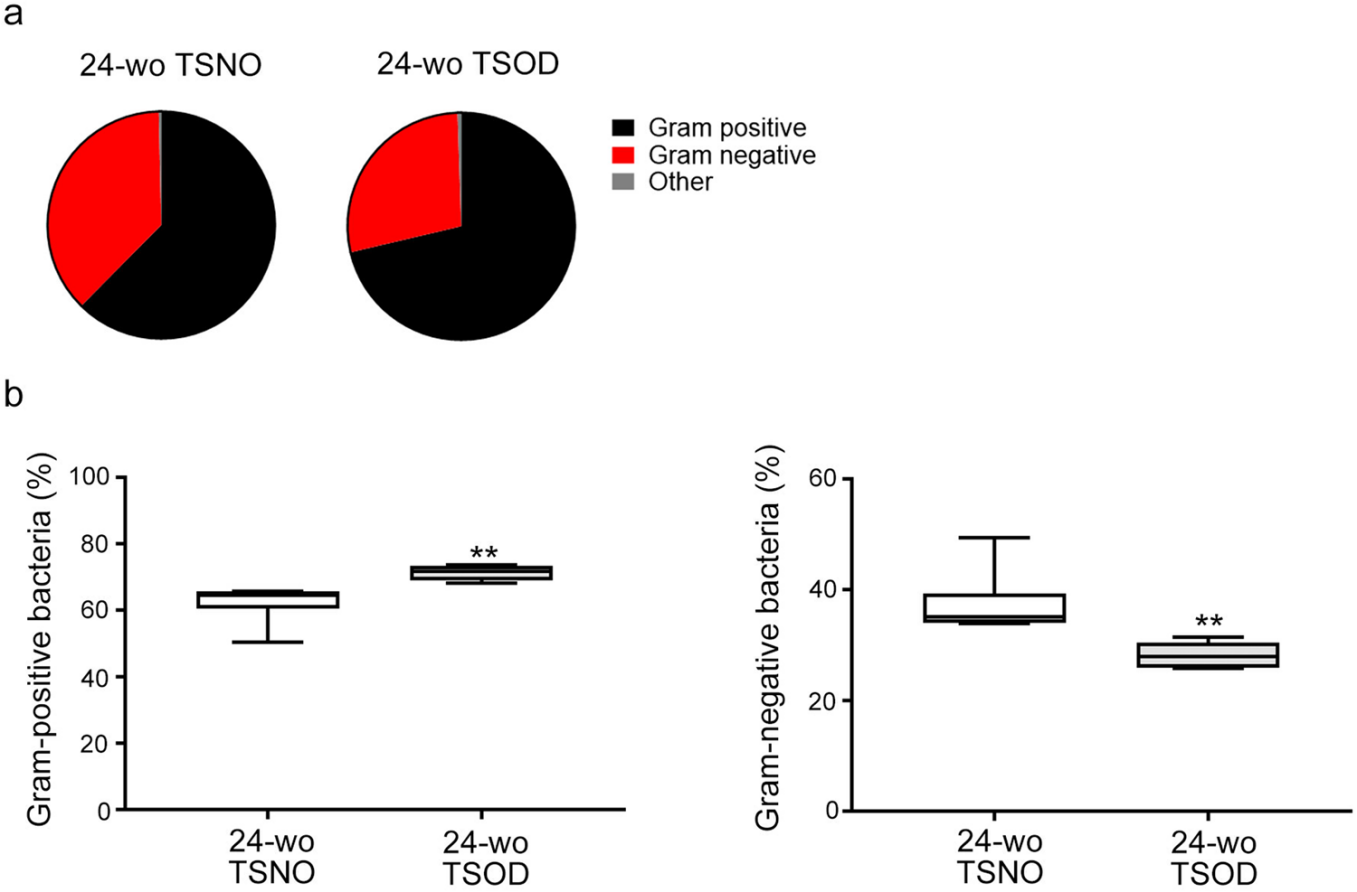
Increased levels of Gram-positive bacteria and decreased levels of Gram-negative bacteria in 24-wk-old (24-wo) TSOD mice. (**a**) Composition of fecal bacteria in 24-wk-old TSOD mice and age-matched TSNO mice. (**b**) Comparison of the percentages of Gram-positive bacteria and Gram-negative bacteria in 24-wk-old TSOD mice and age-matched TSNO mice. Boxes indicate the interquartile ranges between the first and third quartiles, and the lines within the boxes indicate the medians. If no error bars appear, the experimental error was smaller than the symbol itself. **P < 0.01 versus TSNO mice.

**Figure 2:**
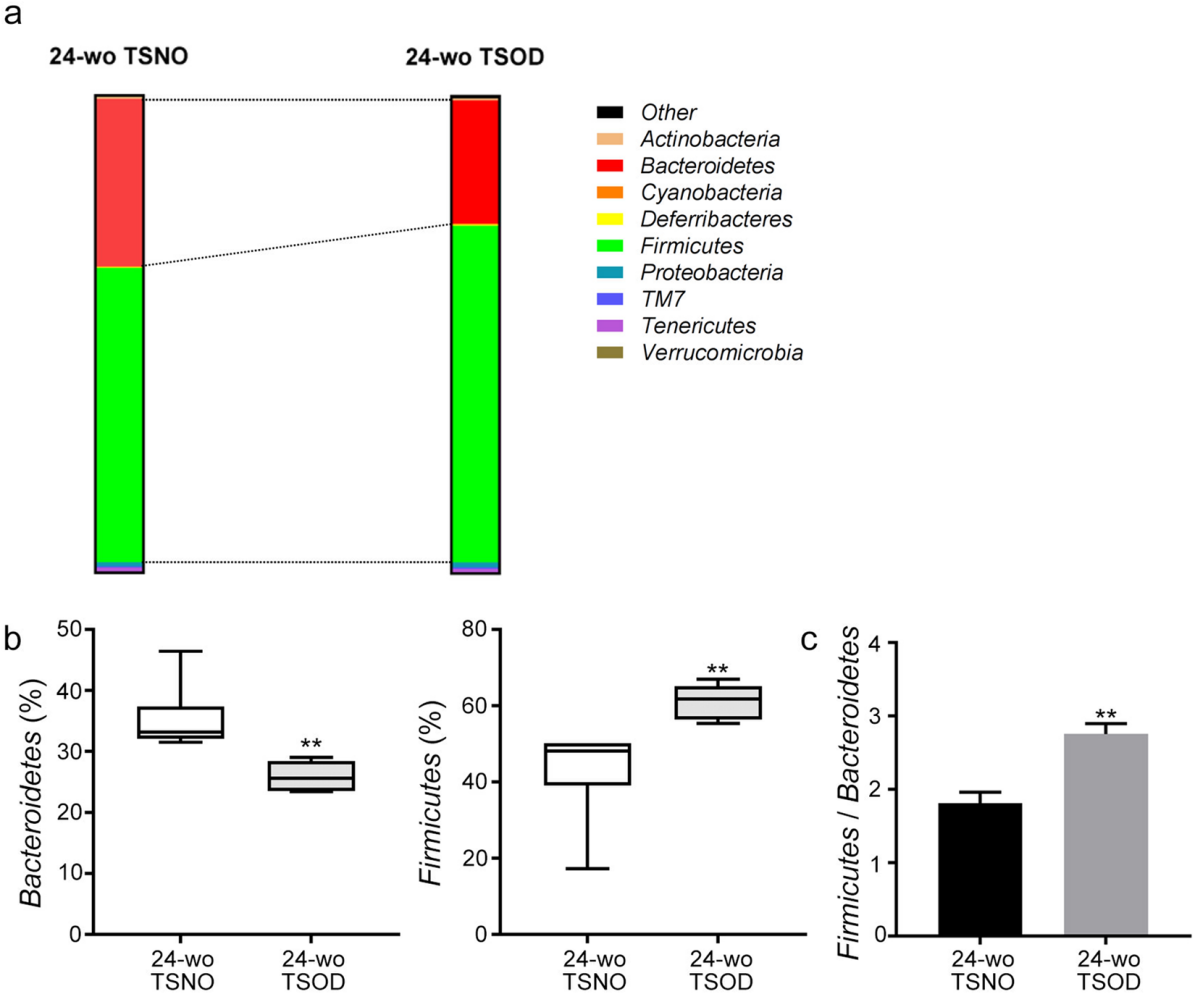
Analysis of fecal bacteria at the phylum level. (**a**) Composition of fecal bacteria at the phylum level in 24-wk-old TSOD mice and age-matched TSNO mice. (**b**,**c**) Comparison of the percentages (**b**) and ratio (**c**) of the obese microbiota Firmicutes and the lean microbiota Bacteroidetes in 24-wk-old TSOD mice and age-matched TSNO mice. (**b**) Boxes indicate the interquartile ranges between the first and third quartiles, and the lines within the boxes indicate the medians. If no error bars appear, the experimental error was smaller than the symbol itself. (**c**) Data are means ± SEM (n = 6). **P < 0.01 versus TSNO mice.

We first confirm that body weights, the ratios of visceral fat or liver to body weight were significantly higher in 24-wk-old TSOD mice (Supplementary Fig. S1a–c). We also scored the liver histology according to our previous report (Supplementary Fig. S1d)^33^. Because microbiota in fecal samples is widely accepted as a surrogate for gut microbiota, we collected feces from 24-wk-old TSOD male mice and Tsumura Suzuki non-obesity (TSNO) mice (controls), and we analyzed the taxonomic compositions of the microbiota by using 16S ribosomal RNA (rRNA) gene sequencing of DNA extracted from the fecal samples. As Fig. 1a shows, 24-wk-old metabolic syndrome-affected TSOD^33^ mice and age-matched control mice manifested different compositions of Gram-positive and Gram-negative bacteria. Compared with control mice, TSOD mice had a significantly higher content of Gram-positive bacteria but a significantly lower content of Gram-negative bacteria (Fig. 1b). We also observed marked changes in the composition of intestinal flora at the phylum level in TSOD mice (Fig. 2a). The percentage of the “obese bacteria”, i.e., Firmicutes, was significantly higher and that of the “lean bacteria”, i.e., Bacteroidetes, was significantly lower in TSOD mice compared with controls (Fig. 2b). These findings were consistent with results from a study of obese human subjects and animals^18,19^. Also, the ratio of Firmicutes to Bacteroidetes was higher in TSOD mice (Fig. 2c). These results reflected the changes in the composition of the populations of Gram-positive and Gram-negative bacteria (Fig. 1), inasmuch as Firmicutes and Bacteroidetes were the most abundant phyla for Gram-positive and Gram-negative bacteria, respectively. These data suggest that the TSOD mouse is a useful model for metabolic syndrome and can manifest the characteristics of the obesity-specific gut microbiome^18,19^.

Also, among the major bacterial families, we found two families in the phylum Firmicutes—Clostridiaceae and Erysipelotrichaceae—that showed significant increases in 24-wk-old TSOD mice (Table 1). These bacteria were reportedly involved in a host’s inflammatory response^35,36^. At the genus level, we detected about 50 bacterial species in both groups of mice. Among these genera, the percentages of nine changed significantly in 24-wk-old TSOD mice (Table 2). As an important finding, these bacteria included *Bilophila*, *Turicibacter*, and *Lactobacillus*, which have been implicated in obesity or NAFLD^37–40^.

**Table 1:** Relative percentages of bacterial groups at the family level that contained more than one genus.

Phylum	Class	Order	Family	TSNO	TSOD
Firmicutes	Clostridia	Clostridiales	Ruminococcaceae	9.858	7.370
				6.925–12.210	6.784–9.423
Firmicutes	Clostridia	Clostridiales	Lachnospiraceae	9.577	5.173
				7.100–10.980	4.179–7.909
Firmicutes	Erysipelotrichi	Erysipelotrichales	Erysipelotrichaceae	2.614	9.891
				1.523–5.542	5.262–20.110^*^
Firmicutes	Clostridia	Clostridiales	Peptococcaceae	0.748	0.500
				0.474–1.386	0.440–0.659
Firmicutes	Clostridia	Clostridiales	Clostridiaceae	0.645	3.476
				0.485–0.947	3.192–4.629^**^
Firmicutes	Clostridia	Clostridiales	Mogibacteriaceae	0.551	0.512
				0.363–0.718	0.454–0.638
Actinobacteria	Coriobacteriia	Coriobacteriales	Coriobacteriaceae	0.299	0.398
				0.227–0.592	0.198–0.564
Proteobacteria	Deltaproteobacteria	Desulfovibrionales	Desulfovibrionaceae	0.065	0.356
				0.020–0.548	0.228–0.419

Values are medians and interquartile ranges. *P < 0.05, **P < 0.01 versus 24-wk-old TSNO mice.

**Table 2:** List of bacteria whose percentages changed significantly in 24-wk-old TSOD mice.

Phylum	Class	Order	Family	Genus	TSNO	TSOD
Bacteroidetes	Bacteroidia	Bacteroidales	Bacteroidaceae	*Bacteroides*	6.350	3.524
					4.374–8.140	3.334–4.001^*^
Firmicutes	Bacilli	Lactobacillales	Lactobacillaceae	*Lactobacillus*	5.629	12.130
					1.817–10.070	11.030–13.240^**^
Firmicutes	Clostridia	Clostridiales	Lachnospiraceae	*Dorea*	0.965	0.434
					0.629–1.316	0.403–0.632^*^
Firmicutes	Erysipelotrichi	Erysipelotrichales	Erysipelotrichaceae	*Allobaculum*	0.521	9.607
					0.078–1.734	4.505–19.320^**^
Firmicutes	Erysipelotrichi	Erysipelotrichales	Erysipelotrichaceae	*Coprobacillus*	0.437	0.130
					0.315–0.650	0.068–0.276^*^
Firmicutes	Clostridia	Clostridiales	Clostridiaceae	*Candidatus Arthromitus*	0.254	0.059
					0.184–0.363	0.011–0.223^**^
Actinobacteria	Actinobacteria	Bifidobacteriales	Bifidobacteriaceae	*Bifidobacterium*	0.004	0.066
					0–0.024	0.013–0.177^*^
Firmicutes	Bacilli	Turicibacterales	Turicibacteraceae	*Turicibacter*	0.000	5.346
					0.000–0.008	4.348–6.740^**^
Proteobacteria	Deltaproteobacteria	Desulfovibrionales	Desulfovibrionaceae	*Bilophila*	0.000	0.169
					0.000–0.000	0.150–0.254^*^

Values are medians and interquartile ranges. *P < 0.05, **P < 0.01 versus 24-wk-old TSNO mice.

### Analysis of the quantity and type of SCFAs in plasma

**Figure 3:**
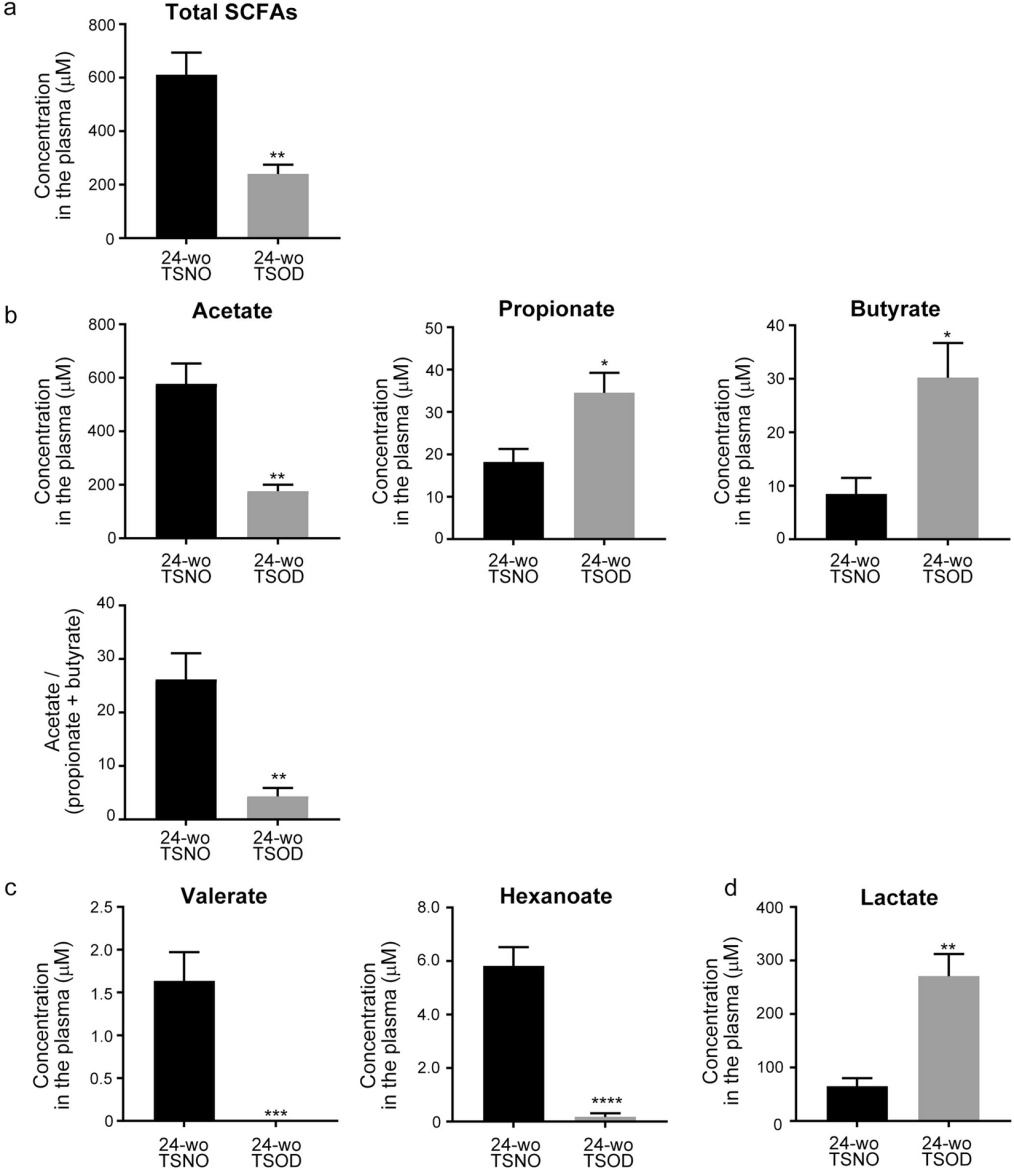
Analysis of plasma SCFAs in TSOD mice and age-matched control mice. (**a**) Total concentration of plasma SCFAs. (**b**) Concentrations of the major SCFAs acetate, propionate, and butyrate, and the ratio of acetate to propionate plus butyrate. (**c**) Concentrations of the minor SCFAs valerate and hexanoate and the precursor of SCFAs (lactate). Data are means ± SEM (n = 6). *P < 0.05; **P < 0.01; ***P < 0.001; ****P < 0.0001.

Whereas the homeostatic balance of gut microbiota benefits a host, an imbalance between beneficial and pathogenic bacteria in the presence of dysbiosis would be detrimental to the host. SCFAs are the main fermentation product of gut microbiota and perform diverse functional roles that affect the host’s physiology^30^. We therefore investigated whether the plasma SCFA profiles changed in TSOD mice. As Fig. 3 shows, we detected acetate, propionate, and butyrate as the major SCFAs, and valerate and hexanoate as the relatively minor SCFAs, in the plasma of TSOD and TSNO mice. The total concentration of plasma SCFAs was significantly lower in 24-wk-old TSOD mice than in the age-matched control mice (Fig. 3a). Acetate, which was the most abundant SCFA, decreased to approximately 30% of the control value in 24-wk-old TSOD mice (Fig. 3b). However, the plasma concentrations of the other major SCFAs—propionate and hexanoate—were 2–3 times higher in 24-wk-old TSOD mice than in controls (Fig. 3b). In agreement with a previous study reporting that leaner people showed a higher ratio of acetate to butyrate plus propionate^41^, the ratio of acetate to butyrate plus propionate in TSOD mice was significantly lower compared with that in controls (Fig. 3b). However, the minor SCFAs valerate and hexanoate, whose plasma concentrations were in the range of 1.5–6.0 nM in TSNO mice, were almost absent in 24-wk-old TSOD mice (Fig. 3c). Furthermore, the plasma concentration of lactate, which is not only the precursor of SCFAs but also a signaling molecule that reportedly affects a host’s physiology by modulating HDAC and G protein-coupled receptor 81 signaling^30^, significantly increased in 24-wk-old TSOD mice (Fig. 3d). These results indicate that the dysbiosis in TSOD mice led to a loss of type and quantity of SCFAs, which may have a role in the development of metabolic syndrome in this mouse model.

## Discussion

To investigate the hypothesis that alterations in the gut microbial community may contribute to the spontaneous development of metabolic syndrome in TSOD mice, we analyzed the gut microbiota and plasma SCFA profiles in 24-wk-old TSOD mice. As expected, we found the following modifications in the microbial community compared with TSNO mice: (1) an increased ratio of Gram-positive bacteria to Gram-negative bacteria, (2) an increased abundance of the obese microbiota Firmicutes and a decreased abundance of the lean microbiota Bacteroidetes, (3) an altered abundance of several bacteria, including *Bilophila*, *Turicibacter*, and *Lactobacillus*, among others (Table 2).

Dysbiosis can generally be categorized into three types: (1) loss of beneficial microbes, (2) excessive growth of harmful microorganisms, and (3) loss of overall microbial diversity^42^. These categories are not mutually exclusive; in most cases, they can occur simultaneously. On the basis of our results described above, we concluded that dysbiosis occurred in 24-wk-old TSOD mice. Gut dysbiosis is defined as qualitative and quantitative changes in gut microbiota, metabolic activity, and local distribution^43^. In addition to having a role in the host’s digestive system, gut dysbiosis has been implicated in the development of obesity and metabolic syndrome by activating a host’s immune response and inflammation, disturbing the intestinal barrier integrity, and causing metabolic abnormalities^44^. The exact role of the gut dysbiosis that we observed here in the etiology or pathology of spontaneous metabolic syndrome in TSOD mice is yet to be elucidated. Colonization experiments such as transfer of microbiota from TSNO mice into TSOD mice or vice versa are required. We previously reported that TSOD mice developed a hepatic tumor that histopathologically resembled human hepatocellular carcinoma (HCC)^45^. In the present study, the percentage of Gram-positive bacteria was significantly higher in 24-wk-old TSOD mice compared with that in controls. As an interesting result, an obesity-associated increase in Gram-positive bacteria in the gut reportedly produced secondary bile acids that promoted HCC development^46^. Bile acids also reportedly activated a signaling network in hepatocytes that triggered hepatic inflammation^47,48^. Given that increased bile acids in the gut are known to favor Gram-positive bacteria^49^, the increase in Gram-positive bacteria in TSOD mice may at least partly contribute to hepatic carcinogenesis. Thus, TSOD mice may be useful for analyzing the effects of novel therapeutic agents, especially those that modulate gut microbiota, for the prevention and treatment of metabolic syndrome-associated HCC.

The gut microbiota was reportedly significantly affected by obesity in humans and in animal models. Ley *et al*. reported that the abundance of Bacteroidetes decreased together with a proportional increase in the phylum Firmicutes in obese mice, compared with their control counterparts, independently of diet^20^. In agreement with this finding, a reduced abundance of intestinal Bacteroidetes associated with an increased abundance of Firmicutes was also observed in obese humans^18^. Also, patients with NASH had a lower percentage of Bacteroidetes compared with both patients with simple steatosis and healthy controls^22^. Although other studies did not always reproduce these results^50,51^, in the present study, the abundance of Firmicutes increased together with a corresponding decrease in Bacteroidetes in the spontaneous mouse model of metabolic syndrome. These alterations in Firmicutes and Bacteroidetes abundance are thought to contribute to the development of obesity by switching the host’s metabolism to increase the adsorption of fatty acids and calories and thereby lead to weight gain^19,52^. Bacteroidetes reportedly increased the production of SCFAs^53^. Thus, the disruption in gut microbiota compositions of Firmicutes and Bacteroidetes in the current study may contribute to the development of obesity in TSOD mice. As an interesting finding, several studies reported an increase in the Firmicutes to Bacteroidetes ratio in patients with irritable bowel syndrome, which shares the characteristic of chronic inflammation with metabolic syndrome^54–56^.

At the microbial family level, we found that the percentages of Erysipelotrichaceae and Clostridiaceae were significantly higher in 24-wk-old TSOD mice than those in controls (Table 1). In other studies, the former increased in patients with type 2 diabetes^57^ and arthritis^58^, both of which are associated with severe inflammation. The latter increased in patients with autism, with unknown mechanisms and functions^59,60^. Bacteria in these families have been implicated in inflammation^35,36^, which suggests that these bacteria may contribute to the pathology in TSOD mice via exacerbation of inflammation. In addition, the percentages of several microbial genera changed in 24-wk-old TSOD mice (Table 2). In agreement with previous studies of patients with NAFLD^39,40^, the percentage of *Lactobacillus* increased in TSOD mice. Inasmuch as this bacterial group can often be used as a probiotic, elucidating the role of *Lactobacillus* in the etiology and pathology of NAFLD or NASH is a future challenge. We found that the percentage of *Turicibacter*, which was implicated in the production of butyrate^61^, was markedly higher in 24-wk-old TSOD mice, in agreement with the increase in plasma butyrate in 24-wk-old TSOD mice. Dimova *et al*. showed that *Turicibacter* increased in mice fed a high-fat diet^38^. However, two studies reported a decrease in *Turicibacter* in mice in response to high-fat feeding^62,63^. Although the role of *Turicibacter* in the development of metabolic syndrome is unknown, it is notable that one study reported an increased abundance of *Turicibacter* in the gut of patients with rheumatoid arthritis, an immune-mediated disease^64^. Another important finding is the presence of the pathobiont *Bilophila* in 24-wk-old TSOD mice, whereas *Bilophila* was absent in control mice. *Bilophila* is a sulfite-reducing pathobiont and causes an interleukin-10-mediated immune response, which leads to colitis in mice^65^. The growth of *Bilophila* can reportedly be promoted by increased taurine-conjugated bile acids^66^, which is consistent with the possibility that bile acid-favoring Firmicutes increased in TSOD mice.

Plasma SCFA profiles in TSOD mice also differed from those in control mice. Compositions of SCFAs depend on the microbial community compositions and the type and quantity of fermentation substrates (i.e., dietary fibers)^37,67,68^. In the present study, because we fed both TSNO and TSOD mice standard chow, the major determinant of plasma SCFAs would supposedly be the composition of gut microbiota. However, because the plasma SCFA profile is an emergent property of the microbial community, making predictions from taxon-based analysis and identifying certain microbes as responsible factors are difficult^69^. Besides acting as an energy substrate, SCFAs can function as signaling molecules by modulating neuroendocrine and anti-inflammatory responses in various tissues and organs^69^. Thus, the type and quantity of SCFAs produced by the gut microbiota are also important for the development of obesity and metabolic syndrome. In the present study, the total SCFA concentrations decreased significantly in 24-wk-old TSOD mice compared with concentrations in controls. With regard to specific SCFAs, the plasma concentration of acetate was significantly lower and concentrations of propionate and butyrate were significantly higher in 24-wk-old TSOD mice than in control mice. Inasmuch as the gut microbiome in TSOD mice favored Firmicutes species, which mainly produce butyrate, and did not favor Bacteroidetes species, which primarily produce acetate and propionate^70^, alterations in the SCFA profiles in TSOD mice seemed to roughly reflect the altered Firmicutes/Bacteroidetes ratio of the microbial community. The mechanism of the increase in propionate in TSOD mice remains to be elucidated, though it may depend on the mouse strains.

Acetate reportedly mediated a *Bifidobacterium*-induced improvement in the intestinal barrier against bacterial endotoxin, possibly by strengthening tight junctions of epithelial cells^71,72^. Although whether the reduced abundance of *Bifidobacterium* in the current study is solely accountable for the decrease in plasma acetate is unknown, that the decrease in plasma acetate may contribute to development of the inflammation-related pathology of metabolic syndrome in 24-wk-old TSOD mice is highly likely. Butyrate has anti-inflammatory activity via modulating HDAC^73^. Propionate also has an anti-inflammatory property^74^. However, Schwiertz *et al*. reported that the propionate level increased in fecal samples of overweight and obese subjects^75^. In the present study, both plasma butyrate and propionate levels increased in 24-wk-old TSOD mice, which strongly suggests that butyrate and propionate may have pathogenic roles via mechanisms yet to be elucidated. We also observed that the level of lactate, the precursor of SCFAs^30^, increased in 24-wk-old TSOD mice, a finding that was consistent with results from previous reports showing that the plasma lactate concentration was higher in subjects with type 2 diabetes and obesity than in normal subjects^76,77^. Given that lactate acts as a mediator of inflammation^78^, increased lactate in TSOD mice may be a pathogenic factor for induction or maintenance of low-grade inflammation. Acetate, which was decreased in TSOD mice, is mainly produced by the Bacteroidetes phylum^70^ of which abundance was also decreased in TSOD mice. As lactate can be further metabolized to acetate^79^, the increase in plasma lactate in TSOD mice might be at least partly due to a decrease in the acetate production by Bacteroidetes. With regard to the minor SCFAs, i.e., valerate and hexanoate, we cannot currently compare our results with others, because of the lack of relevant literature.

In the present study, we observed altered SCFA profiles, with a decrease in total SCFA amounts and diversity, in 24-wk-old TSOD mice. An altered SCFA profile or a disease-specific SCFS profile has been implicated in the pathology of several inflammatory diseases, including Hirschsprung’s-associated enterocolitis^80^, familial Mediterranean fever^81^, and celiac diseases^82,83^. In addition to the respective roles of each SCFA, the total SCFA profile itself, including composition and diversity, may affect a host’s physiology^81,84^. SCFAs reportedly enhanced secretion of glucagon-like peptide (GLP-1) that enhances glucose tolerance^85^. Via the SCFA receptors, SCFAs reportedly modulated the release of proinflammatory cytokines such as tumor necrosis factor α and interleukin 6 that may alter insulin sensitivity and contribute to the development of persistent chronic inflammation^86,87^, which has been implicated in cancer development including that of HCC^88^. It is also reported that gut microbiota suppress fat accumulation via the SCFA receptor^89^. These lines of evidence suggest that the altered SCFA profile observed in TSOD mice potentially contribute to the development of metabolic syndrome and HCC via multiple pathways. Although the physiological and pathological roles of the minor SCFAs are not fully understood, our results suggest that the disrupted plasma SCFA profiles may play a role in the development of obesity and metabolic syndrome in TSOD mice. Additional studies are warranted.

In summary, we observed gut dysbiosis and disruptions in plasma SCFA profiles in TSOD mice. Identification of the mechanisms as well as specific bacteria and bacterial metabolites that are responsible for the pathology and etiology of metabolic syndrome in this mouse model will allow for development of precise treatments to prevent or manage chronic inflammatory diseases. We also propose that the TSOD mouse, which demonstrated its own “microbial signature,” is a useful model to study gut dysbiosis, SCFAs, and metabolic syndrome.

## Methods

### Animals

Six male TSOD mice and six male TSNO mice were purchased from the Institute for Animal Reproduction (Ibaraki, Japan). Two or three mice were reared in plastic cages in a non-barrier-sustained animal room at 23 ± 2 °C in 50 ± 10% relative humidity under a 12/12-h light/dark cycle. All mice were maintained with the basal diet MF (Oriental Yeast Co., Ltd., Tokyo, Japan) and chlorinated water *ad libitum*. Fecal samples were collected from the colon. The study was performed in accordance with the animal experiment guidelines specified by the University of Tokushima. All experimental protocols were approved by the animal research committee of Tokushima University.

### Analysis of the gut microbiome

DNA was extracted from fecal samples by using the isopropanol precipitation technique. Briefly, 30–40 mg of mouse feces was suspended in 19× volume of phosphate-buffered saline and was homogenized by using the FastPrep-24 homogenizer (MP Biomedicals, Santa Ana, CA). A sample consisting of 250 µL of TE buffer (200 mM Tris-HCl, 80 mM ethylenediaminetetraacetic acid, pH 9.0), 50 µL of 10% sodium dodecyl sulfate, 500 µL of TE-saturated phenol (Nippon Gene Co., Ltd., Tokyo, Japan), and 0.3 g of glass beads (diameter 0.1 mm; As-One Co., Ltd., Osaka, Japan, #BZ-01) was added to 200 µL of the ground fecal sample. The fecal samples were further homogenized with a FastPrep-24 homogenizer for 30 s, after which they were centrifuged at 15,000 rpm for 5 min at 4 °C. A sample of 400 µL of a phenol/chloroform/isoamyl alcohol (25:24:1) mixture (Nippon Gene Co., Ltd.) was added to the supernatant, vortexed for 10 s, and centrifuged at 15,000 rpm for 5 min at 4 °C. Isopropanol (250 µL) (Wako Pure Chemical Industries Ltd., Osaka, Japan) was added to 250 µL of the supernatant, mixed by flipping, and kept at room temperature for 10 min followed by centrifugation at 15,000 rpm for 10 min at room temperature. The supernatant was removed, and the resultant pellet was washed with 400 µL of ice-cold ethanol. The extracted DNA was air-dried and then dissolved in 2000 µL of TE buffer (pH 8.0). The V3-V4 region of the bacterial 16S rRNA gene was amplified by using PCR with the TaKaRa Ex Taq HS Kit (TaKaRa Bio, Shiga, Japan) and the primer sets of Tru357F (5ʹ-CGCTCTTCCGATCTCTGTACGGRAGGCAGCAG-3ʹ) and Tru806R (5ʹ-CGCTCTTCCGATCTGACGGACTACHVGGGTWTCTAAT-3ʹ). The DNA was concentrated by amplifying, in triplicate, via PCR: preheating at 94 °C for 3 min, followed by 30 cycles of denaturation at 94 °C for 30 s, annealing at 50 °C for 30 s, extension at 72 °C for 30 s, and a final terminal extension at 72 °C for 5 min. The amplicon was prepared for a sequencing instrument with the method described in a previous report^90^.

### Analysis of SCFAs in plasma

Overall, nine analytes were targeted for SCFA analysis. Acetic acid, lactic acid, propionic acid (PA), butyric acid, isobutyric acid, valeric acid (VA), isovaleric acid (iso-VA), pivalic acid (*tert*-butyl-VA), and caproic acid (CA) were purchased from Wako Pure Chemical Co. For internal standards (IS), PA-*d*_6_, BA-*d*_5_, VA-*d*_9_, and CA-*d*_11_ were obtained from Sigma-Aldrich Co. (St. Louis, MO) and CDN Isotopes Co. (Quebec, Canada). Triphenylphosphine (TPP), 2,2-dipyridyl disulfide (DPDS), and 2-picolylamine were obtained from Tokyo Kasei Co. (Tokyo, Japan). These stock solutions were adjusted by using methanol.

The ultra-performance liquid chromatography (UPLC) system was a Waters Acquity H Class (Waters Co., Milford, MA). A reverse phase analysis was performed via an Acquity UPLC BEH C_18_ column (1.7 μm, 2.1 × 100 mm) at 40 °C. The injection volume was 5 μL. The mobile phase consisting of solvent A (0.1% formic acid in water) and solvent B (0.1% formic acid in methanol) was delivered at a flow rate of 0.3 mL/min. The gradient elution was as follows: B% = 2, 2, 35, 45, and 98 (0, 3, 10, 12, and 14 min). A Waters Xevo TQD triple quadrupole mass spectrometer was operated with an electrospray ionization (ESI) source in the positive mode. The ionization source conditions were as follows: capillary voltage, 2.00 kV; cone voltage, 20–70 V; collision energy, 10–40 eV; source temperature, 150 °C; and desolvation temperature, 400 °C. The cone and desolvation gas flows were 50 and 800 L/h, respectively, and were obtained by using a nitrogen source (N_2_ Supplier Model 24S; Anest Iwata, Yokohama, Japan). On the basis of a previous report for useful derivatization of carboxylic acids^91^, mixed SCFAs and IS solutions were diluted by adding methanol. These solutions were reacted with 2-picolylamine in DPDS and TPP in acetonitrile at 60 °C for 10 min. The reaction mixtures were removed and re-dissolved in 100 μL of methanol/water (80:20, v/v). Finally, the derivatization solutions (5 μL) were analyzed by means of UPLC-ESI-MS/MS. Plasma samples after thawing were added to IS and mixed with equal volumes of methanol and QuEChERS (Supel QuE PSA (EN) 25 mg), vortexed vigorously, and centrifuged at 15,000 rpm for 5 min. The supernatant was then removed, and the remaining residue was re-dissolved in methanol and derivatized by using the process described above for 2-picolylamine. The sample was then analyzed by means of UPLC-ESI/MS/MS.

### Statistical analysis

Values were analyzed via the Mann-Whitney U-test (Figs 1b and 2b, Tables 1 and 2) or unpaired Student’s t-test (Figs 2c and 3), by using Prism software (GraphPad Software, La Jolla, CA). Differences were regarded as significant when *P* < 0.05.

## Electronic supplementary material

Supplementary Information
