# Supplementary material for: Analysis of the gut microbiome and plasma short-chain fatty acid profiles in a spontaneous mouse model of metabolic syndrome
Source: Sci Rep. 2017 Nov 20;7:15876. doi: 10.1038/s41598-017-16189-5 (PMC5696507; doi:10.1038/s41598-017-16189-5)
Supplement: Supplementary file 1 — Supplementary Information [file 41598_2017_16189_MOESM1_ESM.pdf]

Supplementary Information

Analysis of the gut microbiome and plasma short-chain fatty acid profiles in a spontaneous mouse model of metabolic syndrome

Kazuchika Nishitsuji[1], Jinzhong Xiao[2], Ryosuke Nagatomo[3], Hitomi Umemoto[4], Yuki Morimoto[5], Hiroyasu Akatsu[6], Koichi Inoue[3], & Koichi Tsuneyama[5*]

[1]Department of Molecular Pathology, Graduate School of Biomedical Sciences, Tokushima University, 3-18-15 Kuramoto-cho, Tokushima 770-8503, Japan

[2]Next Generation Science Institute, Morinaga Milk Industry Co., Ltd., 5-1-83 Higashihara, Zama, Kanagawa 252-8583, Japan

[3]Laboratory of Clinical and Analytical Chemistry, College of Pharmaceutical Sciences, Ritsumeikan University, 1-1-1 Nojihigashi, Kusatsu, Shiga 525-8577, Japan

[4]Education Support Room for Anatomy, Tokushima University, 3-18-15 Kuramoto-cho, Tokushima 770-8503, Japan

[5] Department of Pathology and Laboratory Medicine, Graduate School of Biomedical Sciences, Tokushima University, 3-18-15 Kuramoto-cho, Tokushima 770-8503, Japan

[6]Department of Medicine for Aging in Place and Community-Based Medical Education, Nagoya City University, Graduate School of Medical Sciences, 1 Kawasumi, Mizuho-cho, Mizuho-ku, Nagoya, Aichi 467-8601, Japan

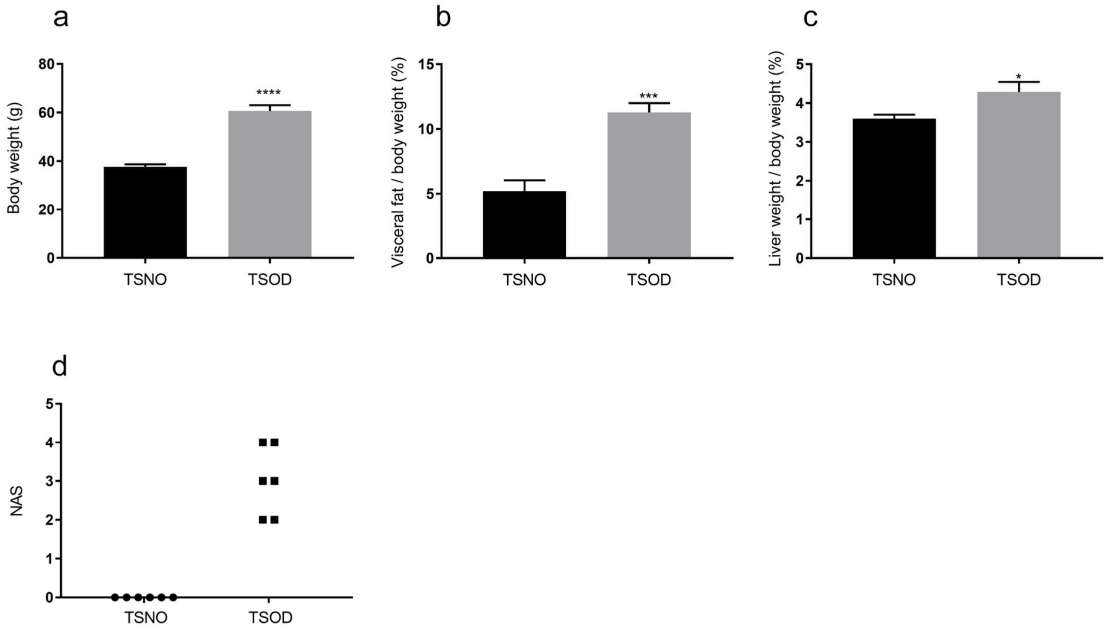

Supplementary Figure S1. General characteristics and NAFLD activity score (NAS) in 24-wk-old TSNO and TSOD mice.

The graphs show body weight **(a)**, visceral fat to body weight ratio **(b)**, liver to body weight ratio **(c)**, and NAS values **(d)** of 24-wk-old TSNO and TSOD mice. Data are means ± SD (a-c). NAS values were calculated as described in the Supplementary Methods (d). $^{*}P < 0.05$; $^{***}P < 0.001$; $^{****}P < 0.0001$ versus TSNO mice by unpaired Student's *t*-test (a-c).

Supplementary Methods

Body weights were recorded at the start and at the end of the experimental period. Immediately after sacrifice, the liver and visceral fat were rapidly excised and rinsed in ice-cold saline. The excised organs were fixed with 10% neutral-buffered formalin and embedded in paraffin for histological analysis. Some of the liver samples were stored at −80 °C. A formalin-fixed, paraffin-embedded liver tissue were processed into 4-mm-thick serial sections and stained with hematoxylin and eosin. A frozen 5-mm-thick section were also stained by using Sudan IV with hematoxylin counterstaining for lipid analysis. Liver histology was scored by using three semiquantitative items: steatosis (0–3), lobular inflammation (0–3), and hepatocellular ballooning (0–2) as described in our previous report[1]. Three representative areas in each section were scored and the averages were used as the final score. The sum of the scores of steatosis, lobular inflammation, and hepatocellular ballooning was presented as the NAFLD activity score (NAS).

Supplementary References

1. Nishida, T. *et al.* Spontaneous onset of nonalcoholic steatohepatitis and hepatocellular carcinoma in a mouse model of metabolic syndrome. *Lab Invest* 93, 230-241 (2013).
